# Ecotoxicological Effects of Conventional and Eco-Friendly Glitter: A Literature Review

**DOI:** 10.3390/biology15110889

**Published:** 2026-06-04

**Authors:** Sara Futia, Paolo Pastorino, Montserrat Solé, Barbara Caldaroni, Rebecca Gentile, Ambrosius Josef Martin Dörr, Marino Prearo, Monia Renzi, Antonia Concetta Elia

**Affiliations:** 1 Department of Chemistry, Biology and Biotechnology, University of Perugia, Via Elce di Sotto 8, 06123 Perugia, Italy; sara.futia@dottorandi.unipg.it (S.F.); bcaldaroni@gmail.com (B.C.); gentile.rebecca@gmail.com (R.G.); ajmartindoerr@libero.it (A.J.M.D.); antonia.elia@unipg.it (A.C.E.); 2 Istituto Zooprofilattico Sperimentale del Piemonte, Liguria e Valle d’Aosta, Via Bologna 148, 10154 Torino, Italy; marino.prearo@izsplv.it; 3 Institute of Marine Sciences (ICM), CSIC, Pg. Marítim de la Barceloneta 37-49, 08003 Barcelona, Spain; msole@icm.csic.es; 4 Department of Life Sciences, University of Trieste, Via Giorgieri 10, 34127 Trieste, Italy; mrenzi@units.it

**Keywords:** aquatic organisms, biodegradable materials, environmental risk assessment, leachates, primary microplastics, terrestrial organisms

## Abstract

Glitter is a distinctive and largely overlooked form of primary microplastic. Unlike more commonly studied microplastics, glitter particles are typically flat, highly reflective, multi-layered, and are composed of polymers such as polyethylene terephthalate, polyvinyl chloride with metallic coatings and a wide range of additives. In response to regulatory restrictions on intentionally added microplastics and increasing consumer demand, “eco-friendly” alternatives based on modified regenerated cellulose, cellulose nanocrystals, or mica have been introduced, although their environmental safety remains insufficiently characterized. This review synthesizes current knowledge on the environmental occurrence and ecotoxicological effects of both conventional and biodegradable glitters. A systematic literature search in Scopus identified 15 peer-reviewed experimental studies meeting predefined inclusion criteria. Evidence spans a wide range of taxa, including bacteria (i.e., *Aliivibrio fischeri*), microalgae and cyanobacteria (i.e., *Phaeodactylum tricornutum*, *Raphidocelis subcapitata*, *Microcystis aeruginosa*), aquatic plants (i.e., *Lemna minor*, *Egeria densa*), marine and freshwater invertebrates as crustaceans (i.e., *Daphnia magna*), bivalves (i.e., *Mytilus galloprovincialis*), sea urchins (i.e., *Paracentrotus lividus*), brine shrimp (*Artemia* sp.) and terrestrial soil fauna (*Eisenia fetida*, *Folsomia candida*). Results indicate that glitter cannot be treated as a uniform stressor: biological responses vary markedly with particle size, shape, colour, polymer type, additive composition, and weathering time, and leachates often exert stronger effects than intact particles. Reported impacts include impaired photosynthesis and growth, oxidative stress, developmental abnormalities, altered energy metabolism, and reduced reproduction. Substantial gaps remain regarding environmental concentrations, ageing processes, mixture effects, and long-term ecological consequences, particularly for biodegradable glitters. Addressing these gaps will require realistic exposure scenarios, mesocosm and field studies, and integrated chemical–biological approaches to support robust risk assessment and safer material design.

## Simple Summary

Microplastic pollution is an emerging issue of growing scientific importance. The literature offers several studies analysing the presence of microplastics and their effects from an ecotoxicological perspective. However, there is one type of microplastic that has received low attention: glitter. Glitter consists of microplastics coated with potentially toxic molecules that can be released into the environment as they degrade. Glitter is commonly associated with special events such as parties, celebrations or cosmetic products but it is a ubiquitous contaminant. It is also present in paints used for cars or boats, or even in fishing bait. Due to their plastic nature, more eco-friendly alternatives were designed and are now available on the market. This review aims to organize and classify all achievable articles on the effects of conventional and eco-friendly glitter on organisms, according to biological classification, trophic level and habitat. The selected articles include alterations in photosynthesis and growth in primary producers, oxidative imbalance, reduced reproduction and tissue damage in invertebrates. The effects are variable depending on the glitter characteristics (polymer type, colour, size) and by whether the exposure involves intact glitter particles or their leachates. The existing literature on the biological effects of both plastic-based glitter and eco-friendly solutions is limited. This highlights the necessity for further investigation, considering the entire ecosystems and more complex environmental scenarios.

## 1. Introduction: Conventional and Ecofriendly Glitter

Microplastics (MPs), plastic particles < 5 mm, are classified into primary and secondary plastics [1,2]. Primary MPs are intentionally produced at these sizes (microbeads present in personal care products such as exfoliants, toothpastes), while secondary MPs are small plastic debris resulting from the fragmentation of larger plastic elements [3,4,5,6]. Secondary MPs can emerge from the action of chemical, physical and/or biological processes in the environment or from anthropogenic action such as MP fragments resulting from the wear of tyres. There is a type of primary MP, that unlike the widely studied microspheres and fragments, remains neglected by the literature although it is found in a variety of products (e.g., nail polishes, accessories, decorations): glitter [7]. The term glitter refers to small, flat, reflective particles [8]. Most of glitter is made from a specific type of polyester (PE) film resulting by stretched polyethylene terephthalate (PET), known as BoPET (Biaxially oriented polyethylene terephthalate), commercially referred to as Mylar^TM^ [8].

Moreover, the literature shows that glitter particles may contain different types of polymers, such as PE, polypropylene (PP), polyvinyl chloride (PVC), and poly (methyl methacrylate) (PMMA), either single or combined. In addition, PVC can present other substances in its composition, such as HCl, benzene, toluene, styrene, and some poly aromatic hydrocarbons (PAHs) like indene, naphthalene, acenaphthene, fluorine, and anthracene [9]. Glitters are usually covered by a metal layer of aluminium, titanium, iron, or bismuth to give them their typical “sparkly” appearance and make their composition more complex than that of the MP particles commonly investigated [10].

Metallic aluminium is obtained from bauxite, a mineral rock. Its extraction generates large amounts of residual waste, which typically contains 15–25% alumina, as well as iron, titanium, and rare earth elements (REEs) at considerable levels, making its disposal challenging [11].

In a study conducted by Yurtsever [12] glitters were treated with an acidic solution (HCl, HNO_3_, H_2_SO_4_), and it was observed that the colour coating, and consequently the metallic coating, dissolved in the solution, whereas the PET substrate remained intact and became transparent. Based on these findings, it can be hypothesized that the metals present in glitters may dissolve in gastric acid upon ingestion.

Furthermore, the composition of glitters depends on manufacturing processes and the pigments used. According to Meirelles et al. [13] glitter contains higher concentrations of metals and metalloids compared to PE and PVC, indicating that many of these elements primarily originate from the coloring stage. Glitters of three colors (green, red, and blue) were characterized. Sixteen elements (Al, As, Au, Ba, Ca, Co, Cr, I, Mg, Mn, Ni, Sb, Sr, Th, V, and Zn) were detected in all three glitter colors. Nine elements (Al, Au, Ba, Ca, Cr, I, Mg, Th, and Zn) were also found in PVC and/or PE samples, suggesting a structural contribution, whereas the remaining elements are most likely attributable to pigments and manufacturing processes. Ti was detected only in red and green glitter samples, with concentrations approximately twofold higher in red glitter (116 mg kg^−1^) than in green glitter (50.4 mg kg^−1^), while it was not detected in blue glitter. Al concentrations were similar in red and blue glitter (5.2 mg·kg^−1^ and 4.9 mg kg^−1^, respectively), but significantly higher in green glitter (19.5 mg·kg^−1^). Some elements were detected exclusively in specific glitter colors, for instance, Nd and Pb were found only in red glitter (4.2 mg kg^−1^ and 0.26 mg kg^−1^, respectively). Cs (0.009 mg kg^−1^) was detected only in blue glitter, whereas K was found only in green (4.99 mg kg^−1^) and red glitter (8.8 mg kg^−1^). These elemental profiles are directly linked to the pigments employed in glitter coloration. For instance, green pigments are commonly derived from glauconite or celadonite, which are minerals that represent a major source of K [13].

Commercial glitter ranges in size from 50 to 6350 μm, but the most common size is around 200 μm [14]. Glitter can be produced in a variety of shapes, usually as in precision-cut pieces of uniform size, sometimes with notches. The most common shapes include hexagonal, square, triangle, stripe, heart, star, crescent moons, diamond, rhombus, flower, snowflake, butterfly, irregular forms [8]. Despite the countless shapes, the most commercially available ones are those most easily recognizable, such as circles, stars, and crescent moons [15,16]. Glitter is entirely man-made. It may be tiny pieces of aluminium foil or plastic with a vapor-deposited aluminium layer, or it may consist of multiple layers of plastic with no metal layer at all. In the manufacturing process, before it is cut into individual tiny particles, it is presented in the form of rolled sheets of foil or plastic. Most often the sheets are cut into regular geometric shapes that allow a two-dimensional surface to be completely filled without producing any waste. It is assumed that glitter particles are present only in rare events: parties, celebrations, demonstrations, or in cosmetics and personal care products [7]. However, glitter is widely used in a variety of products, including arts and crafts (e.g., glitter glue, paints, decorative paper), decorations and ornaments (e.g., Christmas decorations, greeting cards), clothing and accessories (e.g., apparel, bags, shoes), packaging and wrapping materials (e.g., cosmetic, confectionery, or toy packaging), and paints and specialty coatings (e.g., boat and automotive paints, body paints). Moreover, glitter is often used in fishing lures, and companies sell glitter containing dough as well as gels into which lures may be dipped [16].

Glitter easily disperses in the environment. According to Reininger et al. [17], glitter reaches distances from 12 to 261% greater than spheres of equivalent volume. This highlights that glitter has a high potential for atmospheric transport, which is in turn influenced by its settling behaviour and physical properties (e.g., size and shape) [17]. Furthermore, due to their small size and light weight, glitter is easily transferred and retained upon contact, often without notice, which renders it key evidence in criminal investigations [15,16]. Glitter particles can be released into water bodies directly or indirectly. The former occurs when glitter particles are washed away during makeup or glittery body paint removal, or when MPs are released following outdoor recreational activities [10]. Glitter particles are small enough to bypass the drum screen (1.5 mm) in wastewater treatment plants (WWTPs) during the primary treatment stage, partitioning preferentially to sludge due to the density of common glitter polymers such as polybutylene terephthalate (PBT) and polyethylene terephthalate (PET) [18]. Indeed, glitter accounted for approximately 24% of microplastics in waste activated sludge and remained detectable in treated effluent, demonstrating that WWTPs can act both as sinks and secondary sources of glitter contamination [18]. Current evidence indicates that glitter is already present in a wide range of environmental matrices, including lake and beach sediments, river sediments, sewage sludge, wastewater effluents, street dust, urban atmospheric deposition, and beach sands (Table 1). Reports from Canada, Norway, Iran, Finland, the United Kingdom, Australia, and Brazil demonstrate that glitter contamination is geographically widespread and occurs in both aquatic and terrestrial environments [18,19,20,21,22,23,24]. Notably, the detection of glitter particles in urban dust and atmospheric fallout suggests that atmospheric transport may represent an important but understudied dispersal pathway, whereas their occurrence in sediments and wastewater-derived sludge indicates long-term environmental accumulation. Increased glitter contamination observed in beach sediments following large recreational events further highlights the direct contribution of human activities to environmental releases. Nevertheless, compared with other microplastic categories, the occurrence and abundance of glitter remain poorly characterized, and available data are limited to a small number of studies and regions. Consequently, the environmental distribution of glitter is likely underestimated due to methodological constraints, inconsistent particle classification, and the frequent inclusion of glitter particles within broader categories such as fragments or films [8].

On 17 October 2023, the Commission Regulation (EU 2023/2055) restricted the use of MPs, including glitters, in cosmetics and construction materials. Thus, companies already consolidated in the market, and new ventures have developed more sustainable alternatives to conventional glitter. These alternatives are mainly composed of different types of natural products and are estimated as less harmful to the environment [25].

In response to the demand for “eco-friendly” materials, new products have been designed and developed to be more environmentally friendly, as an alternative to conventional glitter which are constitute mostly by PET and PVC polymers. Thus, biodegradable glitter derives from regenerated cellulose, modified regenerated cellulose (MRC) mainly from Eucalyptus trees or from the natural mineral mica [26]. However, there is a more ethical choice to mining mica, namely the production of a synthetic alternative: fluorphogopite. Mica particles present in cosmetics can be coated with metal oxides (as titanium dioxide, iron oxides) of different types and thicknesses, allowing for the creation of different shades of a given colour [16]. Furthermore, unlike conventional glitter, the shape of the mica particles is completely irregular and random, although they fall within a range [27]. Glitter widespread use, high dispersal potential, and structural and chemical complexity, which distinguish it from other MPs, make a dedicated synthesis of its ecotoxicological relevance increasingly necessary. Glitter is now recognized as a relevant source of MP contamination, entering the environment through domestic wastewater and via particles shed from skin and other surfaces that accumulate on land and are subsequently transported into aquatic systems by stormwater runoff [10]. Once released, glitter behaves similarly to other MPs, posing both physical and chemical hazards: ingestion may obstruct digestive or respiratory structures in aquatic organisms [28], while additives and embedded metals can leach into surrounding waters and induce toxic effects [29]. Accordingly, the present review aims to synthesize current evidence on the environmental occurrence of glitter, evaluate the effects of both conventional and biodegradable glitters across aquatic and terrestrial organisms, and identify key research gaps that must be addressed to support robust environmental risk assessment, regulatory frameworks, and the development of safer technological alternatives.

## 2. Methodology

The Scopus database https://www.scopus.com/ (accessed on 20 March 2026) was used to retrieve relevant literature, with no restriction on publication year and coverage extended through December 2025. The search query included the terms: “glitter” AND “ecotoxicology” OR “ecotoxicity” OR “toxicology” OR “toxicity” AND “effects”. To ensure scientific rigor, explicit inclusion and exclusion criteria were applied.

Inclusion criteria: (i) peer-reviewed original research articles; (ii) written in English; (iii) studies evaluating the biological or ecotoxicological effects of glitter particles; (iv) studies conducted on any aquatic or terrestrial organism (including microorganisms, plants, invertebrates, and vertebrates); and (v) studies providing quantitative or qualitative biological endpoints (e.g., growth, mortality, reproduction, photosynthesis, oxidative stress).

Exclusion criteria: (i) reviews, meta-analyses, conference abstracts, thesis, book chapters and editorials; (ii) studies focusing exclusively on analytical methodologies or MP detection without biological testing; (iii) studies addressing non-glitter MPs; and (iv) papers lacking sufficient methodological detail to extract ecotoxicological endpoints.

**Figure 1:**
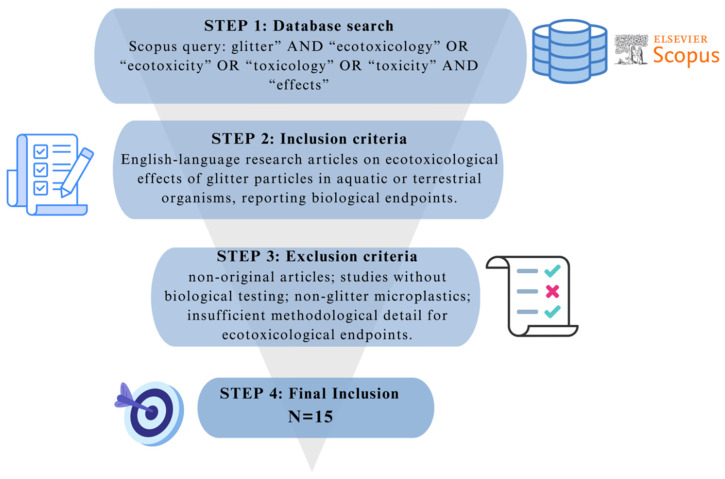
Workflow of the literature screening and selection process used in this review, showing the Scopus database search, inclusion and exclusion criteria, and final selection of studies on the ecotoxicological effects of glitter particles (N = 15).

Following screening and full-text evaluation, 15 studies fulfilled former criteria and thus, were included in this review (Figure 1). From each article, the following information was extracted: year of publication, model organism, glitter colour and shape, glitter type (conventional or eco-friendly), particle size, exposure conditions, measured endpoints, and observed effects (Table 2).

## 3. Ecotoxicological Effects of Glitter

The available literature on the effects of glitter exposure has been predominantly conducted in aquatic environments, encompassing marine [9,10,29,30,31,32,33], freshwater [6,26,35,36], and terrestrial [37,38] ecosystems. Only two studies have simultaneously addressed more than one ecosystem type [14,34] (Table 2). Glitter colour was reported in most cases, with multi-coloured particles being the most frequently tested, followed by white and silver glitters, whereas colour information was missing in a limited number of studies (Table 2). Particle shape was less consistently described: when reported, glitters were mainly characterized by regular polygonal forms (e.g., star, hexagon, rectangle, pentagon), while several studies did not provide explicit shape information (Table 2). Regarding polymer composition, PET-based glitters were the most commonly used, either alone or in mixtures, followed by acrylic-based materials (PMMA, MA-VC, methyl acrylate), while cellulose-derived materials, mica-based glitters, and BoPET were investigated in fewer studies; in some cases, glitters were generically described as non-biodegradable without polymer specification (Table 2). Particle size was explicitly reported in 12 studies, spanning a wide range from ~2–6 µm at the lower end to ~3000 µm at the upper end; however, most experiments focused on micro-sized glitters between approximately 241 and 1035 µm, with several studies testing size classes up to 2000–3074 µm (Table 2). Exposure concentrations were almost exclusively expressed as mass-based metrics, with 14 studies using mg/L or mg/kg. In aquatic systems, tested concentrations generally ranged from 0.01–0.1 mg/L at the lowest levels up to 350–500 mg/L, while terrestrial studies employed substantially higher doses, reaching 7500–21,000 mg/kg. Only one study adopted particle number concentrations (12.5–25 particles/L), highlighting the strong predominance of mass-based exposure approaches in current glitter research (Table 2). In the following subsections, the ecotoxicological effects reported in these studies are presented and discussed separately for marine, freshwater, and terrestrial ecosystems.

### 3.1. Ecotoxicity in Marine Organisms

#### 3.1.1. Bacteria

In ecotoxicological testing, *Aliivibrio fischeri* is widely used as a standard organism to assess acute toxicity via bioluminescence inhibition in both marine and freshwater matrices [39]. Although it is a marine bacterium, it is frequently applied in freshwater bioassays; for consistency, it is therefore discussed here within this marine section.

Piccardo et al. [14] investigated the toxicity of leachates derived from nine glitter types differing in shape, size, colour, and polymer composition (PMMA, PE, PA). Glitter particles were immersed in artificial seawater and freshwater for 3, 90, and 180 days to generate percolates. Acute toxicity tests were conducted after 15 and 30 min of exposure to seawater leachates prepared at 90% strength, using a 15% bioluminescence inhibition threshold as the reference effect level. At the earliest leaching stage (3 days), *A. fischeri* showed significant inhibition (31.3–61.2%) when exposed to leachates from decorative PMMA glitters, all hexagonal and approximately 221.6 µm in size, differing only in colour (green, purple, orange, pink, and yellow). After 90 days of immersion, responses remained below the effect threshold, indicating a reduction in acute toxicity at this intermediate stage. After 180 days, however, responses diverged according to glitter characteristics: leachates from two PMMA glitters (orange and pink), identical in composition, shape, and size, again caused marked inhibition (37.2% and 79.3%, respectively), whereas biostimulation was observed for two silver glitters differing in polymer type (PE-based paint glitter and PMMA-based hair gel glitter) and particle size (244.7 µm and 954.1 µm), with bioluminescence increases of 27.2% and 15.1%, respectively.

Freshwater toxicity was assessed using lyophilized *A. fischeri* (Microbiotests Inc., Belgium Kleimoer 15, 9030 Gent, Belgium, Germany), exposed for 15 and 30 min to leachates obtained by immersing 100 mg of each glitter type in 1 L of ISO freshwater. Toxic effects were defined using the same 15% inhibition threshold [40], and test validity was confirmed using 3,5-dichlorophenol as a positive control. After 3 days of immersion, leachates from a decorative-art glitter (pink, PMMA-based, hexagonal, 221.64 µm) induced bioluminescence inhibition of 21.3%, exceeding the reference threshold. Although immersion time did not consistently influence toxicity, several PMMA-based glitters of identical size but different colours produced effects above the threshold. Overall, these results demonstrate that specific glitter leachates can impair *A. fischeri* bioluminescence under freshwater conditions.

Across the entire ecotoxicological battery, *A. fischeri* emerged as the second most sensitive trophic level. Responses were generally stronger in saltwater than in freshwater and showed no consistent relationship with polymer type. The more pronounced effects observed in saltwater are likely due to the higher presence of ions and salts compared to freshwater, which may promote a greater release of chemical compounds or interact more strongly with them, resulting in enhanced effects relative to the freshwater matrix. Although the effects observed in freshwater were less pronounced than those in the saltwater matrix, the pink PMMA polymer also induced effects under freshwater conditions, indicating a substantial release of chemical additives likely associated with the glitter coloration. Multivariate analyses highlighted that leachate toxicity depends on species sensitivity, immersion duration, and exposure medium, underscoring the chemical complexity of glitter-derived leachates [14].

The effects of biodegradable glitter leachates on *A. fischeri* were subsequently examined by Doval-Miñarro et al. [30]. Acute toxicity tests were performed using commercially available biodegradable chunky glitters (MoonTM Creations, London, UK), pink and gold in colour, hexagonal in shape, and ranging from 0.5 to 1 mm in size. Depending on colour, particles contained aluminium powder (CI 77000), Red 7 (CI 15850:1), or Yellow 5 Lake (CI 19140:1). Leachates were generated by immersing glitter particles for 96 h in both type II laboratory water and natural seawater collected from the Mediterranean Sea off the southeastern coast of Spain, the former representing abiotic degradation processes and the latter more environmentally realistic conditions. *A. fischeri* was exposed for 15 min to blanks and serial 1:2 dilutions of the leachates. No toxic effects were observed under any exposure condition. Although slightly higher responses were recorded for leachates obtained in purified laboratory water, these effects were insufficient to calculate EC50 values, indicating an overall lack of acute toxicity [30]. The absence of toxic effects observed may be attributed to the use of biodegradable glitters, which lack the plastic core typically containing plastic additives that may induce toxic effects.

#### 3.1.2. Invertebrates

A growing set of recent laboratory studies has begun to address gaps regarding glitter sources, inputs, environmental fate, and ecotoxicological impacts in marine fauna. Experiments exposing planktonic crustaceans (*Artemia* spp.) to intact particles and associated leachates have shown clear toxic responses. That is, *Artemia* sp. exposed for 48 h to methyl acrylate-vinyl chloride (MA–VC) glitter displayed significant mortality at 0.1 mg/L (LOEC) with an LC50 of 0.35 mg/L. In this study, microscopy analysis revealed particle aggregation on appendages and metallic fragments in the gut [29]. Similar effects occurred in *A. salina* exposed to metallic-coated PET glitter, which exhibited intestinal lesions, digestive tract accumulation, and reduced survival (LD50 = 14.78 mg/L). Further developmental assays demonstrated suppressed hatching efficiency, with pronounced inhibition at ≥10 mg/L and with the associated Al leached from this PET glitter reaching ~0.3 ppm within 48 h, exceeding EPA water quality thresholds, and likely contributing to impair cysts development [31]. In these cases, glitter particles can be considered dual-mode contaminants, capable of inducing both physical/mechanical stress (e.g., intestinal lesion) and chemical toxicity through the release of additives, pigments, and metal coatings.

Embryotoxicity tests using sea urchins (*Echinometra lucunter*, *Arbacia lixula*, and *Paracentrotus lividus*) indicated species-specific sensitivity to glitter dispersions and leachates. Green glitter, chemically more complex and containing 12 compounds including BHT, propylparaben, and PVC-associated volatiles was generally more toxic than MA–VC-based white glitter [9]. Dose–response relationships were apparent, although mildly bimodal patterns emerged for some species. EC50 values for *E. lucunter* were lower for green glitter than for white glitter, consistent with higher chemical load. Toxicity Identification and Evaluation (TIE) procedures implicated volatile substances, oxidants, and metals as major drivers of toxicity. In another study, leachates from PMMA, PE, and PA glitter types tested on *P. lividus* produced limited effects at early immersion times but caused developmental delays and abnormalities after 90–180 days, underscoring the importance of prolonged environmental exposure [14].

Toxicity tests were performed on eggs of the sand dollar *M. quinquiesperforata*, which is abundant on the sandy beaches of neotropical and subtropical regions. Exposure to green glitter with particle sizes between 0.002 and 0.006 mm did not show statistically significant results; on the contrary, a significant response was observed when this species was exposed to white glitter particles with sizes between 0.06 and 2 mm, but without a dose–response relationship. In addition, LOEC values were 0.01% and 100% in the case of exposure to green particles, while they were between 0.01% and 0.1% in the case of white glitter dispersion. The different toxicities of the two glitter dispersions may be due to the fact that the two contaminants have different hydrophilicity, with white glitter being more easily mixed in water [9].

Filter-feeding bivalves exhibited even greater sensitivity. Embryos of *Perna perna* exposed to glitter dispersions developed abnormally at concentrations < 10 mg/L, with green glitter again producing the strongest effects. In *Mytilus galloprovincialis*, 7-day exposures to various glitter types revealed size-dependent particle retention: smaller particles accumulated in tissues, whereas larger or star-shaped particles were less efficiently filtered. Biomarker analyses (SOD, GPx, MDA) showed significant oxidative responses, especially following exposure to smaller glitter particles, while GST remained unaffected. Particle length was inversely related to recovery in water, suggesting preferential retention of smaller particles in the digestive tract [10]. It is difficult to distinguish whether the observed effects are primarily attributable to stress induced by the physical particles, to the release of chemical additives, or to a synergistic combination of both mechanisms.

Leite et al. [33] investigated the effects of glitter exposure across a salinity gradient using three shrimp species with different ecological niches. The marine shrimp *Penaeus vannamei*, one of the most widely cultivated species worldwide [41], was exposed for 10 days to white glitter particles (0.08 mm) at concentrations of 0, 0.4, 4, and 40 mg/L under salinities of 20, 30, and 35 S. Pyrolysis–mass spectrometry identified several potentially hazardous compounds in the glitter, including benzene, anthracene, methyl acrylate, and toluene. Limited mortality was observed (one individual at 4 and 40 mg/L). Glitter exposure induced marked physiological alterations, including increased oxygen consumption, particularly at higher salinities, and extreme increases in ammonia excretion under specific conditions. Energy metabolism shifted from protein oxidation in controls to mixed protein–lipid utilization in exposed shrimp. Additional effects included changes in hepatosomatic index, increased haemolymph osmolality, and severe histopathological damage to gills and intestinal tissues. Although oxygen consumption recovered after transfer to glitter-free water, several parameters, including hepatosomatic index, osmolality, and tissue structure, showed incomplete recovery.

The diadromous shrimp *Macrobrachium amazonicum* was tested under freshwater and salinities of 10 and 20 S using the same glitter concentrations. No mortality occurred; however, a strong reduction in oxygen consumption (≈70%) was observed at 10 S, along with increased ammonia excretion at higher glitter concentrations. Glitter exposure altered energy substrate utilization and caused a substantial reduction in haemolymph osmolality at 40 mg/L, indicating impaired osmoregulatory capacity.

The exclusively freshwater shrimp *Macrobrachium potiuna* showed greater tolerance to glitter exposure, with no mortality and only moderate physiological effects. Oxygen consumption decreased by approximately 20% compared to controls, while ammonia excretion increased notably at the lowest glitter concentration tested. Energy metabolism remained protein-based across all treatments, and haemolymph osmolality was unaffected.

Overall, the study demonstrates that glitter exposure can disrupt respiration, nitrogen excretion, energy metabolism, osmoregulation, and tissue integrity in crustaceans, with effects strongly modulated by species ecology and salinity. Marine and diadromous species were generally more sensitive than strictly freshwater taxa, highlighting the importance of considering salinity gradients when assessing the ecotoxicological risks of glitter particles.

#### 3.1.3. Microalgae and Cyanobacteria

Glitter particles can also interfere with marine primary producers by altering light availability through shading or reflection. The filamentous cyanobacterium *Nodularia spumigena* was exposed for 21 days to five concentrations of non-biodegradable glitter (0–350 mg/L) to assess growth and physiological responses [34]. Optical density measurements indicated maximum growth at 100 mg/L, while concentrations ≥ 137.5 mg/L reduced cell density, with the highest concentration yielding the lowest biomass. Growth rates generally did not differ from those of the control, except on day 21; however, low coefficients of determination (R2 < 0.7) suggested inconsistent growth patterns, likely due to complex cell-glitter interactions, such as aggregation, reflection of light, or physical interference.

Cyanobacteria can exhibit both inhibitory and stimulatory (hormetic) responses to abiotic stress. At 350 mg/L, *N. spumigena* showed a pronounced increase in cell volume, reflecting a stress-related resistance strategy. Larger biovolume can result from reduced division and metabolite accumulation, decreasing the surface-to-volume ratio and thereby limiting exposure to contaminants. No statistically significant changes were detected for chlorophyll-a or total carotenoids, although carotenoid concentrations declined slightly at 200 and 350 mg/L. Cells exposed to 350 mg/L also exhibited altered chlorophyll fluorescence, including loss of chlorophyll II signal, alongside increased fragmentation of trichomes. Overall, the effects were dose-dependent, impairing growth and altering cellular morphology.

Toxicity of the nine glitter types of leachates previously described was also evaluated in the diatom *Phaeodactylum tricornutum*, a standard species in marine bioassays. Nine commercial glitter types varying in shape, size, colour, and polymer composition (PMMA, PE, PA) were immersed in artificial seawater for 3, 90, and 180 days to generate percolates. Growth inhibition after 72 h (3 days) was quantified spectrophotometrically, with a 10% change used as the threshold for biological relevance. Responses were highly variable and frequently displayed hormesis. Growth inhibition reached up to 52.2% in some treatments, whereas most percolates induced biostimulation. The strongest biostimulatory effect (38.2%) occurred following exposure to the 180-day leachate from a silver, hexagonal PMMA glitter type. These findings demonstrate that glitter-derived chemicals can influence microalgal physiology in both inhibitory and stimulatory directions, depending on exposure duration, polymer type, and additive release [14].

### 3.2. Ecotoxicity in Freshwater Organisms

#### 3.2.1. Invertebrates

The cladoceran *Daphnia magna*, a freshwater crustacean commonly used in toxicity testing, was exposed to leachates derived from the nine glitter types previously examined by Piccardo et al. [14]. Leachates prepared after three immersion periods produced only occasional effects, mainly after longer immersion times. Overall, *D. magna* exhibited low sensitivity to glitter leachates and ranked among the least affected organisms in the ecotoxicological test battery, together with sea urchins [14].

In parallel, the increasing use of bioplastics has raised interest in the ecological impacts of alternative glitter materials. Green et al. [26] exposed the invasive mud snail *Potamopyrgus antipodarum* to conventional oil-based glitter and several biodegradable glitter types for 36 days. Four silver-coloured glitters were tested, differing in material composition, PET, modified regenerated cellulose (MRC), natural mica, and synthetic mica, as well as particle size. Freshwater mesocosms were established with river sediment, and each received 50 adult snails. At the end of the exposure period, mesocosms treated with MRC glitter displayed roughly twice the number of individuals compared with the controls and PET treatments. This increased abundance likely reflects the species’ high reproductive rate, which contributes to its success as an invasive organism [26].

#### 3.2.2. Plants

In the former mesocosm experiment by Green et al. [26], the effects of glitter were also assessed on the duckweed *Lemna minor*, collected from the River Glaven (UK). Each mesocosm received 500 plants and, after a 48 h acclimation, 500 mg/L of the glitter types previously described. Glitter settled onto the water surface within 24 h. After 36 days, plants were collected, counted, and analysed for root length and chlorophyll content. Although biomass, plant number, and chlorophyll concentrations did not differ between treatments, root lengths were approximately twice as long in control mesocosms compared to those exposed to PET, modified cellulose, or synthetic mica glitter, indicating that both conventional and “eco-friendly” glitters reduced root development in *L. minor* [26]. This may be because even glitter materials described as “eco-friendly” are still coated with a metallic layer, typically aluminium, which is phytotoxic to plants [42].

In another study, interactions between glitter and aquatic macrophytes were further examined in *Egeria densa*, a key species in lentic and lotic systems. Yoshida et al. [35] exposed apical fragments of *E. densa* to green commercial glitter (0.04 g/L) under light and dark conditions to evaluate photosynthetic performance. Net and gross photosynthesis were measured using the light–dark bottle method, and a P–I experiment [43] assessed light–photosynthesis relationships. Glitter reduced both net and gross photosynthesis, with net photosynthesis 1.54 times higher in the absence of glitter. Light measurements indicated that glitter particles lowered incident radiation inside the bottles, consistent with their reflective metallic surface. As light availability is a key limiting factor for macrophytes, reduced irradiance explains the observed decrease in photosynthetic efficiency. Glitter presence also reduced respiration rates, although less markedly, suggesting additional stress mechanisms such as oxidative effects.

The P-I curves showed neither saturation nor photoinhibition in control or glitter treatments, but the half-saturation constant increased by 36.84% in the presence of glitter, indicating that *E. densa* required more light to achieve half-maximum photosynthesis. Because *E. densa* is an ecological keystone species, contributing to oxygen production, habitat structure, and food resources, a reduction in its photosynthetic performance may have broader implications for ecosystem functioning and could trigger trophic cascade effects [35].

#### 3.2.3. Microalgae and Cyanobacteria

Both conventional and “eco-friendly” glitters can affect freshwater primary producers. In mesocosms, Green et al. [26] measured chlorophyll a, b, and c in the water column after exposure to PET, MRC, natural mica, and synthetic mica glitters. Chlorophyll a did not differ across treatments, but chlorophyll b and c were approximately three times higher in control mesocosms than in any glitter treatment, suggesting reduced biomass of green microalgae, diatoms, and dinoflagellates, key primary producers. A decline in phytoplankton biomass was attributed to leachates containing aluminium and acrylic coatings [26].

Toxicity to *Raphidocelis subcapitata* was evaluated by Piccardo et al. [14] using 72 h exposure to leachates from nine glitter types (UNI EN ISO 8692:2012). Growth inhibition stayed within effect thresholds after 9 days but increased after 180 days, with some treatments causing biostimulation (15.1–20.8%), indicating hormesis. Responses varied: most leachates inhibited growth, while two PMMA-based glitters (pink and yellow, hexagonal, ~221.64 µm) produced mild stimulation. Photosynthetic primary producers were the most sensitive trophic level, responding with either reduced or enhanced growth depending on exposure time and leachate composition. Changes in leachate chemistry over time, such as increased release of trace metals, may have influenced these effects [14].

Combined effects of glitter particles and their leachates have rarely been examined. Wang et al. [28] exposed *Desmodesmus* sp. to suspensions and leachates of PET glitters of five colours (silver, black, red, green, blue; ~150 µm). Suspended glitter increased turbidity and reduced photosynthetic fluorescence parameters (ΦPSII, α, rETRmax) by 4.9–6.2%, indicating interference with light utilization. PET glitter alone caused no detectable effects on growth or chlorophyll.

In contrast, leachates of red and green PET glitters significantly inhibited growth (12.5%) and chlorophyll content (8.1%), while silver, black, and blue leachates had no measurable impact. Fluorescence parameters confirmed photosynthetic disruption specifically for red and green leachates [36].

Chemical analyses showed that leachates contained metals (Mg, Al, Cr, Zn, Sr, Sb, Ba) and organic additives. Red leachates had the highest concentrations of toxic metals (e.g., Cr, Ba, Sr, Sb) and the greatest diversity of organic contaminants (dimethyl phthalate, butyl lactate, octamethylcyclotetrasiloxane, isobutanol, hydroxy(dimethyl)silane, 1-butanol). Regression and PCA linked algal inhibition to combinations of these metals and organics, highlighting chemical composition as the primary toxicity driver [36].

When *Desmodesmus* sp. was exposed to mixtures of glitter particles and their corresponding leachates, red and green mixtures caused the strongest effects: reduced growth (14.4%), increased SOD activity, inhibited CAT and POD activity, and elevated MDA content (150.3%), indicating oxidative stress. Mixtures of silver, black, and blue glitters with their leachates produced negligible responses. The IBRv2 index ranked toxicity of mixtures as: red > green > black ≈ blue > silver, and cluster analysis grouped red/green mixtures separately from the less toxic silver/black/blue mixtures [36]. Overall, phytoplankton studies show that glitter leachates are generally more harmful than glitter particles alone, and glitter colour, reflecting differences in pigment composition and additive content, is a critical determinant of toxicity.

Biodegradable glitter was also tested on the bloom-forming freshwater cyanobacterium *Microcystis aeruginosa* [34]. Growth peaked at 50 mg/L glitter, whereas exposure to 200 mg/L produced the lowest biomass. At 200 and 350 mg/L, carotenoid concentrations declined by 0.25 μg/mL, and cell biovolume increased in a dose-dependent manner. Despite these stress responses, *M. aeruginosa* accumulated more chlorophyll-a than the control, suggesting enhanced light-harvesting capacity and a degree of resistance to glitter-induced toxicity [34].

### 3.3. Ecotoxicity in Terrestrial Invertebrates

Studies on terrestrial organisms are scarcer compared to those on aquatic species, but available work indicates that glitter can negatively impact soil fauna. Trakić et al. [37] exposed the earthworm *Eisenia fetida* to commercial glitters of various shapes (hexagonal, square, rectangular; 75–200 µm) mixed into artificial soil at 2.5%, 5%, and 7% (*w*/*w*). After 28 days, mortality increased at 5% and 7%, and worms exhibited lethargy at higher concentrations. Growth responses varied, with reduced weight gain at 2.5% and 5% and an apparent increase at 7%, likely reflecting glitter retention rather than true growth. Avoidance behaviour showed no clear preference for control or glitter-treated soil. Fluorescence imaging confirmed glitter accumulation in the oesophagus and gizzard, with higher loads in the 7% treatment. The presence of particles in the digestive tract and increased fluorescent signal intensity indicate ingestion and possible obstruction, explaining impaired condition and altered behaviour [37].

Biodegradable glitter has also been investigated as a potential alternative to conventional PET glitter. Green et al. [26], Machado et al. [34], and Chen et al. [38] assessed whether plant-based or cellulose-derived formulations reduce ecological risk in terrestrial models in their respective studies. Chen et al. [38] exposed the springtail *Folsomia candida* to PET glitter and to untreated and heat-treated cellulose nanocrystal (CNC) glitters for 28 days. PET glitter (silver, hexagonal, ~100 µm) and CNC glitters (~64–177 µm, multi-coloured, irregular) were added to soil at 10, 100, and 1000 mg/kg. Survival remained high (82–96%) and adult body length showed no differences between treatments. However, reproduction was significantly reduced at 1000 mg/kg PET glitter (61% fewer juveniles relative to controls), whereas untreated and heat-treated CNC glitters did not affect reproduction at any concentration. These findings indicate that CNC glitter poses lower ecological risk than PET glitter and that heat treatment does not increase toxicity [38].

### 3.4. Preliminary Risk Tiering Framework for Glitter Materials

A preliminary hazard-based risk tiering framework can be proposed based on polymer composition, additive content, environmental persistence, and the ecotoxicological responses reported in the literature. Following polymer hazard classification approaches developed by Lithner et al. [44], which ranked polymers according to the hazards associated with their constituent monomers and identified polyvinyl chloride (PVC) among the most hazardous conventional polymers, acrylic- and vinyl-based formulations (e.g., methyl acrylate-vinyl copolymer, MA-VC, and PVC-containing glitters) may be classified as high-concern materials due to their association with mortality, developmental abnormalities, physiological disruption, and the potential release of hazardous monomers and degradation products [44]. PMMA-containing glitters may be classified as moderate-to-high concern, as polymethyl methacrylate is produced from comparatively more hazardous monomers than polyolefins and because toxicity may be amplified by the presence of pigments, plastic additives, and metallic coatings [44]. PET- and BoPET-based glitters can be considered of moderate concern because, despite being derived from less hazardous monomers than PVC, they remain highly persistent in the environment and have been associated with oxidative stress, tissue damage, and ecological effects linked to additive release and contaminant interactions [44,45]. PE-based glitters may be provisionally classified as low-to-moderate concern, with risks primarily associated with particle ingestion, physical interactions, environmental persistence, and contaminant transport rather than intrinsic polymer toxicity [44]. Finally, cellulose-derived alternatives, including modified regenerated cellulose (MRC) and cellulose nanocrystal (CNC) glitters, may be considered lower-concern materials because of their biodegradable nature; however, recent evidence suggests that biobased and plant-derived materials can still release chemically complex mixtures and should therefore not be assumed inherently safe without dedicated ecotoxicological [46]. Overall, because glitter toxicity is strongly influenced by additives, pigments, metallic coatings, and other leachable substances, future risk assessment frameworks should integrate both polymer-specific hazard rankings and additive-related toxicity rather than relying solely on polymer identity [47,48].

## 4. Conclusions

This review highlights glitter as a distinctive yet still insufficiently investigated component of microplastic contamination. Unlike the microspheres and fragments that dominate microplastic research, glitter is defined by a multi-layered architecture, reflective surfaces, and chemically complex formulations that include polymers, metallic coatings, and diverse additives. These characteristics confer exceptional environmental mobility and interaction potential and clearly demonstrate that glitter cannot be treated as a uniform or simplistic contaminant. Moreover, as a type of microplastic, conventional glitter has the ability to adsorb both organic and inorganic contaminants and therefore act as a chemical carrier [49]. Instead, its ecotoxicological effects arise from the combined influence of particle morphology, polymer type, additive chemistry, environmental transformation processes, and species-specific sensitivity, resulting in highly variable biological responses. For instance, chromatic differences in glitter are often associated with qualitative and quantitative variations in their chemical composition, influencing their toxicological potential.

Despite the limited number of available studies, consistent evidence indicates that glitter can disrupt key biological functions across marine, freshwater, and terrestrial ecosystems. Observed effects include impaired photosynthesis and growth in primary producers, oxidative imbalance and metabolic disruption in invertebrates, reduced reproduction, and tissue damage, collectively pointing to the potential for glitter to interfere with fundamental ecosystem processes. Primary producers often emerge as particularly sensitive targets, raising concerns about bottom-up effects that may propagate through food webs.

At the same time, major knowledge gaps constrain robust environmental risk assessment. First, most experimental designs rely on short-term exposures to pristine particles, whereas environmental glitter is subject to photodegradation, fragmentation, biofilm colonization, and chemical aging, processes known to modify buoyancy, surface reactivity, bioavailability, and toxicity but rarely incorporated into ecotoxicological testing [50]. Second, organisms in natural systems are exposed simultaneously to particles, leachates, and weathering products under multiple interacting stressors (i.e., ultraviolet radiation, temperature and salinity variability, and fluctuating dissolved organic matter) that are seldom replicated in laboratory studies [51]. Third, the pronounced influence of glitter colour, shape, polymer composition, and additive load challenges the notion of glitter as a single contaminant class. Despite this complexity, no classification framework currently exists to group glitter types for regulatory or risk-assessment purposes, and standardized analytical methods for detecting and quantifying glitter in environmental matrices are still lacking. As a result, global occurrence patterns remain largely unknown, particularly in soils, sediments, and atmospheric deposition.

The growing availability of biodegradable and “eco-friendly” glitters represents an important technological response to regulatory restrictions. Initial evidence suggests that some alternatives based on modified regenerated cellulose, cellulose nanocrystals, or mica may pose lower ecological risks than conventional polyethylene terephthalate- or polyvinyl chloride-based glitters. However, their environmental fate, degradation products, and long-term ecological interactions remain poorly understood, especially under realistic environmental conditions, and should not be assumed benign without rigorous testing.

Looking forward, glitter research exemplifies the need for a broader shift in microplastic ecotoxicology, from short-term, organism-centered assays toward ecosystem-relevant approaches. Future studies should prioritize long-term mesocosm and field experiments that integrate realistic concentrations, environmental ageing, hydrodynamic transport, trophic transfer, and interactions with co-occurring pollutants [52]. Atmospheric transport and deposition, already shown to be more efficient for glitter than for spherical microplastics, represent an especially underexplored pathway with potential significance for both terrestrial and freshwater ecosystems.

Regulatory frameworks will need to evolve accordingly, accounting for colour- and additive-specific toxicity, prolonged leachate release, and the heightened sensitivity of primary producers. Future research should aim to develop standardized methods for the sampling, identification, classification, and chemical characterization of glitter and, more broadly, microplastics. Currently, indeed, multiple methodological approaches exist that are not always directly comparable, which limits the interpretation and consistency of results. Addressing these challenges will require coordinated efforts that integrate environmental chemistry, ecotoxicology, materials science, and policy development. Only through such cross-disciplinary collaboration can the ecological footprint of glitter be accurately assessed and effectively mitigated in the coming decade.

## Figures and Tables

**Table 1:** Occurrence of glitter particles in environmental matrices reported in the scientific literature.

Location	Environmental Matrix	Glitter Occurrence	Main Findings	Reference
Lake Ontario, Canada	Lake and beach sediments	Glitter particle identified among environmental microplastics	One of the earliest reports explicitly identifying glitter in environmental sediments	Ballent et al. [19]
Norway	Sewage sludge	Glitter represented ~1.7% of microplastics in sludge	Demonstrated accumulation of glitter in wastewater sludge	Lusher et al. [20]
Tehran, Iran	Urban deposited dust	Glitter-like particles detected in atmospheric fallout	Evidence of atmospheric transport of glitter-derived plastics	Dehghani et al. [21]
Finland	WWTP effluent and receiving waters	Glitter particles observed throughout treatment stages	WWTPs act as pathways and partial sinks for glitter particles	Lares et al. [22]
United Kingdom	River sediments	Glitter particles reported qualitatively	Demonstrated occurrence of glitter in freshwater sediments	Hurley et al. [23]
Newcastle, Australia	Raw wastewater, waste activated sludge, treated effluent	Glitter constituted ~24% of MPs in sludge and 2.41% in effluent	First study specifically investigating the fate of glitter during wastewater treatment	Raju et al. [18]
Rio de Janeiro, Brazil	Beach sand	Glitter and glitter-derived fragments increased after Carnival celebrations	Direct release of cosmetic glitter to coastal environments	Sodré et al. [24]

**Table 2:** Summary of experimental studies investigating the effects of glitter particles on marine, freshwater, and terrestrial organisms. For each study, it is reported the test organism, glitter colour, particle shape, polymer composition, particle size range (µm), exposure concentration (expressed as mass-based units or particle number where applicable), the main biological effects observed, and the corresponding reference. Abbreviations: PMMA, polymethyl methacrylate; PE, polyethylene; PA, polyamide; PET, polyethylene terephthalate; MA-VC, methyl acrylate-vinyl copolymer; MRC, modified regenerated cellulose; CNC, cellulose nanocrystals; BoPET, biaxially oriented polyethylene terephthalate; LOEC, lowest observed effect concentration; NOEC, no observed effect concentration; EC50, median effective concentration; LC50, median lethal concentration; LD50, median lethal dose; SOD, superoxide dismutase; GPx, glutathione peroxidase; MDA, malondialdehyde; GST, glutathione S-transferase.

Organism	Glitter Color	Glitter Shape	Glitter Polymer	Size (µm)	Concentration	Effects	Major Findings	Reference
**Marine**								
*Aliivibrio fisceri*	Gray, yellow, pink, orange, violet, green pink, gold	Star, hexagon, rectangle, pentagon hexagon	PMMA, PE, PA, PET modified regenerated cellulose	241.3–3073.8 500–1000		31.3–61.2% bioluminescence inhibition; hormesis (in marine medium); 21.3% bioluminescence inhibition (in freshwater medium); No effects		Piccardo et al. [7] Doval-Miñarro et al. [30]
*Artemia* sp.	White		MA-VC	0.06–500			0.1 mg/L LOEC; LC50 48 h 0.35 mg/L	Abessa et al. [29]
*Artemia salina* Brine shrimp	Silver		PET		10, 50, 100 mg/L	Lesions in the intestine; accumulation in the digestive tract;	14.78 mg/L LD50	Das Pramanik et al. [31]
*Echinometra lcunter* Rock urchin	Green, white		MA-VC	2–6, 60–2000			EC50, NOEC, LOEC 272.2, 200, 300 mg/L respectively (white) 105.9, <50, 50 mg/L respectively (green)	Abessa et al. [32]
*Arbacia lixula* Black sea uchin	Green		MA-VC	2–6, 60–2000			EC50, NOEC, LOEC 246.1, 100, 200 mg/L respectively	Abessa et al. [32]
*Paracentrotus lividus* Purple sea uchin	Gray, yellow, pink, orange, violet, green	Star, hexagon, rectangle, pentagon	PMMA, PE, PA, PET	241.3–3073.8		developmental delays and abnormalities		Piccardo et al. [7]
*Mellita quinquiesperforata* Keyhole sand dollar	White, green		MA-VC	2–6, 60–2000	0.01, 0.1, 1, 10, 100 mg/L	morphological anomalies; alteration/delay in development	0.01 mg/L LOEC;	Albanit et al. [9]
*Perna perna* Brown mussel	Green, white		MA-VC	2–6, 60–2000		abnormalities in embryonic development at concentrations < 10 mg/L	EC50, NOEC, LOEC NC, <10, 10 mg/L (white) respectively 23, <10, 10 mg/L (green) respectively	Abessa et al. [32]
*Mytilus galloprovincialis* Mediterranean mussel	Gray, yellow, pink, orange, violet, green	Star, hexagon, rectangle, pentagon	PMMA, PE, PA, PET	241.3–3073.8	12.5, 25 particles/L	Smaller particles accumulation; SOD, GPx, MDA significant oxidative responses, GST unchanged		Provenza et al. [10]
*Penaeus vanamei* Whiteleg shrimp	white		methyl acrylate	80	0, 0.4, 4, 40 mg/L	Mortality; oxygen consumption increase; ammonia excretion increase; mixture of proteins and lipids as energy source; gill lamellae bifurcation, detachment of the lamellar epithelium, interstitial edema, intestinal mucosa rupture, muscle tissue rupture, submucosal tissue increase		Leite et al. [33]
*Macrobrachium amazonicum* Amazon River prawn	white		methyl acrylate	80	0, 0.4, 4, 40 mg/L	No mortality; oxygen consumption decrease, 70%; ammonia excretion increase; hemolymph osmolality reduced		Leite et al. [33]
*Macrobrachium potiuna* Brazilian freshwater prawn	white		methyl acrylate	80	0, 0.4, 4, 40 mg/L	No mortality; decrease in oxygen consumption, 20%; ammonia excretion increase; hemolymph osmolality not affected		Leite et al. [33]
*Nodularia spumigena* Cyanobacteria			non-biodegradable	277–1035	0–350 mg/L	Max growth at 100 mg/L; reduced cell density > 137.5 mg/L		Machado et al. [34]
*Phaeodactylum tricornutum* Marine pennate diatom	Gray, yellow, pink, orange, violet, green	Star, hexagon, rectangle, pentagon	PMMA, PE, PA, PET	241.3–3073.8		highly variable responses; hormesis;		Piccardo et al. [7]
**Freshwater**								
*Daphnia magna* Water flea	Gray, yellow, pink, orange, violet, green	Star, hexagon, rectangle, pentagon	PMMA, PE, PA, PET	241.3–3073.8		low sensitivity;		Piccardo et al. [7]
*Potamopyrgus antipodarum* New Zealand mud snail	Silver		PET, MRC, mica and synthetic mica		500 mg/L	Growth rate increase;		Green et al. [26]
*Lemna minor* Common ducweed			PET		10, 100, 1000 mg/L	root length increase;		Boots et al. [6]
*Egeria densa* Brazilian waterweed	Green		non-biodegradable		40 mg/L	Net (1.5-fold) and gross photosynthesis reduction		Yoshida et al. [34,35]
*Raphidocelis subcapitata* Green algae	Gray, yellow, pink, orange, violet, green	Star, hexagon, rectangle, pentagon	PMMA, PE, PA, PET	241.3–3073.8		15.1% growth inhibition, 20.8% biostimulation;		Piccardo et al. [7]
*Desmodesmus* sp. Green algae	Silver, black, red, green, blue		PET	150	50 mg/L	4.9–6.2% reduction photosynthetic fluorescence parameters (ΦPSII, α, rETRmax) SOD activity increased; CAT and POD activity inhibited, 150.3% MDA content; 12.5% growth inhibition; 8.1% chlorophyll content		Wang et al. [36]
*Microcystis aeruginosa* Cyanobacteria			non-biodegradable	277–1035	0, 50, 100, 200, 350 mg/L	Growth peak at 50 mg/L; lowest biomass at 200 mg/L; 0.25 μg/mL carotenoids decreased at 200–350 mg/L; dose-dependent increase cell biovolume		Machado et al. [34]
Phytoplankton	Silver		PET, MRC, mica and synthetic mica		500 mg/L	no change in chlorophyll a; twofold increase in chlorophyll c in PET glitter–treated samples		Green et al. [26]
**Terrestrial**								
*Eisenia fetida* Red wiggler worm		Hexagonal, square, rectangular	BoPET		7500, 15,000, 21,000 mg/Kg	mortality increased at 5% and 7%; lethargy at higher concentrations; particle presence in digestive tract		Trakić et al. [37]
*Folsomia candida* White springtail	Silver, multicolored	Hexagonal, irregular	PET, CNC	~100, ~64–177		82–96% survival; no differences between treatments in adult body length; 61% reduced reproduction at 1000 mg/Kg PET glitter		Chen et al. [38]

The bold text (marine, freshwater, terrestrial) is used to indicate the habitat-based classification of the organisms included in the study.

## Data Availability

No new data were created or analyzed in this study.
